# Patient- and Family-Centered Care for the Emergency Admission of a Child with Autism Spectrum Disorder

**DOI:** 10.14789/ejmj.JMJ25-0029-CR

**Published:** 2026-02-03

**Authors:** AMANE ENDO, AKIO NAKAMURA, MAYU NAKAGAWA, MITSUYOSHI SUZUKI, TOSHIAKI SHIMIZU

**Affiliations:** 1 Department of Pediatrics, Juntendo University Faculty of Medicine, Tokyo, Japan; 1 Department of Pediatrics, Juntendo University Faculty of Medicine, Tokyo, Japan

**Keywords:** patient- and family-centered care, autism spectrum disorder, nephrotic syndrome, pediatric emergency admission

## Abstract

Patient- and Family-Centered Care (PFCC), an evolving healthcare model, places patients and their families at the center of care, promoting collaboration between healthcare providers and families. We report the case of a 5-year-old boy with autism spectrum disorder (ASD) who required emergency hospitalization for nephrotic syndrome. Severe anxiety and behavioral difficulties hindered routine medical procedures. Applying the PFCC approach, a multidisciplinary team―including a child psychiatrist and child life specialist―collaborated closely with the family to develop a personalized care plan based on detailed developmental history and behavioral assessment. Strategies included positive language, visual schedules, and stuffed animals to demonstrate procedures, thereby reducing anxiety and improving cooperation. These interventions enabled the patient to adapt to the hospital environment, comply with treatment, and achieve remission after one month. Post-discharge follow-up revealed continued medication adherence. The family’s involvement was crucial, enhancing communication and ensuring consistent care. The PFCC approach not only improved the boy’s medical outcomes but also fostered trust and collaboration. The case emphasizes the importance of adopting PFCC, especially for children with developmental disabilities like ASD, to provide compassionate and effective care, ensuring long-term success. As the prevalence of developmental disabilities rises, the PFCC model will become increasingly essential in healthcare, being of significance not only in pediatric care but also for all medical professionals.

## Introduction

Patient- and Family-Centered Care (PFCC) is an evolving approach to healthcare that places the patient and their family at the very core of the treatment process. Traditional care models often regard the patient as a passive recipient of medical care, whereas PFCC emphasizes collaboration and partnership between healthcare providers and the families they serve. The patient and their family are not just subjects of care; they are integral members of the healthcare team. In this model, medical personnel act as facilitators or supporters, ensuring that care is both effective and aligned with the patient's and family's preferences and values.

The Institute for Patient- and Family-Centered Care (IPFCC) has been a strong advocate for this approach, promoting the idea that healthcare should be delivered "with" patients and families, rather than "to" or "for" them. This collaborative model not only enhances the quality of care but also fosters trust and communication between healthcare providers and patients. The American Academy of Pediatrics (AAP) has underscored the importance of PFCC in a statement titled “Patient- and Family- Centered Care and the Pediatrician’s Role^1)^.” This statement highlights the necessity of integrating PFCC into pediatric practice, particularly in settings where the patient is a child with developmental disabilities such as Autism Spectrum Disorder (ASD)^2)^. The prevalence of ASD and other developmental disorders has been steadily increasing in recent years, requiring medical professionals to adapt their practices accordingly^3)^.

Children with developmental disabilities, including those diagnosed with ASD, present unique challenges in clinical settings, especially during emergency admissions. These children often have difficulty understanding and coping with new and stressful situations, such as hospitalization, which can exacerbate their symptoms and lead to behavioral challenges. In these cases, a PFCC approach is not just beneficial but essential to ensure that the child receives the best possible care while minimizing the stress and anxiety associated with hospitalization.

This case report presents the emergency admission of a 5-year-old boy diagnosed with ASD who developed nephrotic syndrome, necessitating urgent care in the pediatric department. Due to his severe anxiety about hospitalization, a PFCC-based approach was adopted, involving close collaboration with the patient's parents and a multidisciplinary team. Reports that describe PFCC in detail in the context of pediatric renal disease are limited; this case adds concrete ward-level implementation steps for a child with ASD. We reported here due to its importance in hospital management of ASD patients, whose numbers have been increasing in recent years.

## Case presentation

The patient, a 5-year-old boy diagnosed with ASD, was attending a rehabilitation facility designed to support children with developmental disabilities. His parents had noted significant changes in his physical condition approximately ten days before his admission to the hospital. Specifically, the boy had gained 3 kilograms, representing about 13% of his total body weight—a concerning sign that warranted further investigation. Three days before admission, the boy developed edema in his scrotum, penis, and lower extremities, which further alarmed his caregivers and led to his initial visit to the outpatient clinic.

During the outpatient visit, the patient underwent a series of tests to determine the cause of his symptoms. The results revealed a urinary protein/creatinine ratio (UP/UC) of 36.13 g/gCre, and his serum albumin level was dangerously low at 0.9 g/dL. These findings confirmed the diagnosis of nephrotic syndrome, a condition that required immediate hospitalization for close monitoring and treatment.

However, the hospitalization presented significant challenges, primarily due to the patient's ASD and his intense anxiety about medical procedures. The boy exhibited extreme reluctance to undergo medical examinations, including blood sampling, and displayed behavioral issues such as tantrums and difficulty cooperating with healthcare providers. These behaviors indicated his difficulty in adapting to the hospital environment and his inability to follow the daily schedule of hospital life, which is often rigid and unfamiliar to children with ASD.

Recognizing the need for a specialized approach, the pediatric team quickly expanded to include a child psychiatrist and a child life specialist. These professionals conducted a detailed developmental assessment to better understand the patient's needs and how best to support him during his hospital stay.

## PFCC approach

Through the application of the PFCC approach, the healthcare team developed a comprehensive care plan that emphasized collaboration among all staff members and focused on understanding the feelings and needs of both the patient and his family.

**Figure 1:**
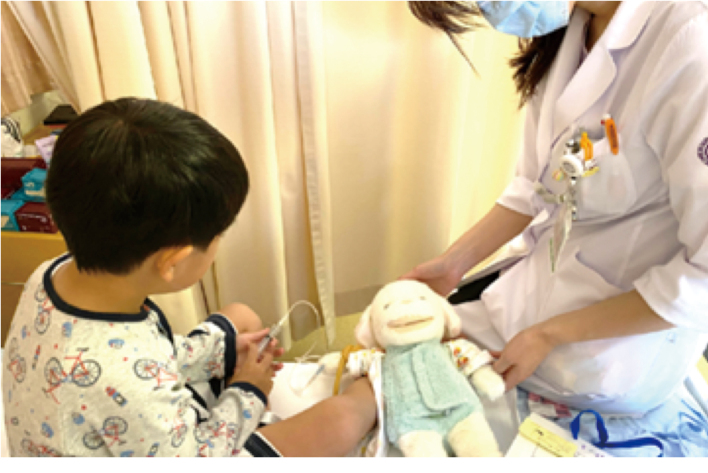
Preparation with stuffed animals Medical procedures were demonstrated on stuffed animals to reduce anxiety and help the child cooperate with interventions.

Day 1 (admission): The patient initially refused blood sampling. A child life specialist intervened and introduced rehearsal of the procedure using a stuffed animal, which gradually reduced resistance (Figure 1).

Day 2: A structured interview with the family and standardized cognitive and developmental assessments were conducted. Based on these results, individualized behavioral support strategies were formulated.

The first step in this process was to conduct a thorough re-interview with the family to obtain a detailed history of the patient’s upbringing and development. This interview revealed key characteristics of the patient's ASD, such as difficulties with eye contact and a lack of interest in other people during early childhood. These insights were crucial for tailoring the care plan to the patient's specific needs.

Further assessments, including the Tanaka-Binet Intelligence Quotient (IQ) test, revealed that the boy had an IQ 71, borderline range of intellectual functioning. Additionally, the Pervasive Developmental Disorders Autism Society Japan Rating Scale (PARS-TR) revealed a peak score of 23 during early childhood, indicating the severity of his ASD symptoms. With this information in hand, the team devised strategies to support the patient in ways that were consistent with his developmental level and behavioral characteristics. One of the key strategies involved using specific language and communication techniques tailored to the patient's needs. For example, the team used positive rephrasing without negative words to reduce the boy's anxiety during medical procedures. As a result, administration of sedatives or anxiolytics to the pediatric patients was unnecessary.

**Figure 2:**
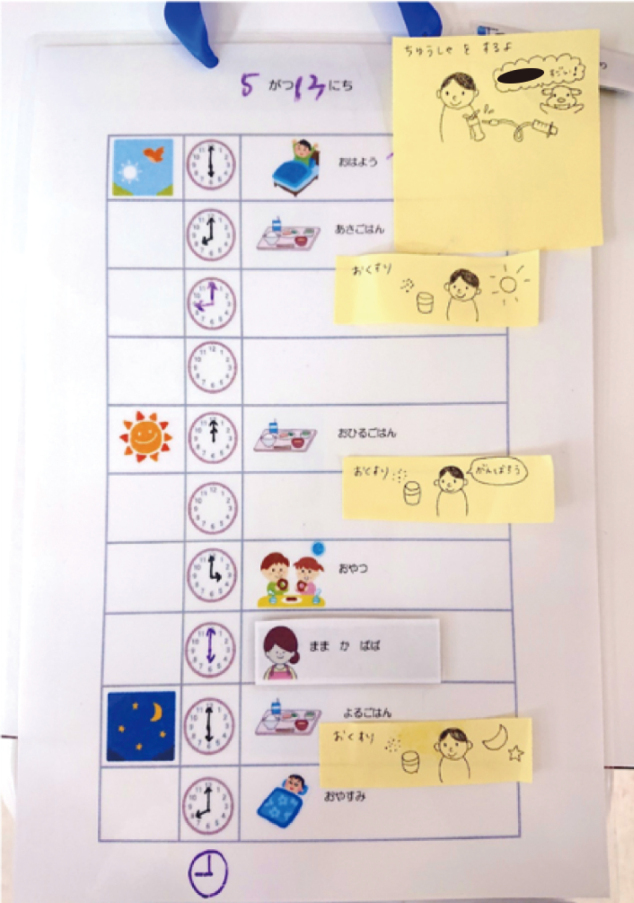
Visualized daily schedule A pictorial daily schedule illustrated routine activities, enabling the child to anticipate events and adjust to hospital life.

Day 3: We created visual schedules to help the boy understand and anticipate the daily routine, which is often a source of stress for children with ASD (Figure 2). A visual daily schedule was introduced, which helped the patient anticipate events and cooperate with the morning blood sampling.

Week 2: Pediatric nurse specialists applied tailored approaches during meals and medication times, leading to improved acceptance of food intake and oral medication.

**Figure 3:**
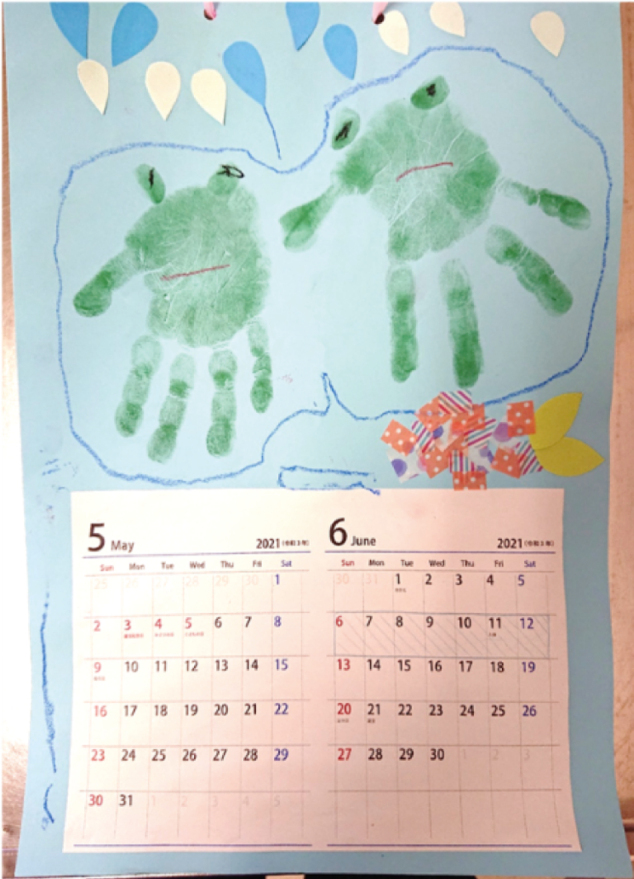
Visualized monthly schedule for hospitalization A calendar-style schedule outlined the overall treatment and discharge plan, supporting family understanding and adherence.

Week 4: Prior to discharge, the patient and family rehearsed the home routine using the visual schedule, which supported good adherence to medications after discharge (Figure 3).

These efforts were not limited to the medical team; they were coordinated and shared with the entire multidisciplinary team, including the child's parents, child life specialists, residents, nurses, ward nursery staff, and even nursing students. This comprehensive approach ensured that everyone involved in the boy's care was on the same page and that the patient received consistent and supportive care throughout his hospital stay.

## Outcomes

The PFCC approach led to significant improvements in the patient's ability to adapt to the hospital environment and cooperate with medical procedures. Over time, the boy became more comfortable with the daily routine and less resistant to medical tests and treatments. His nephrotic syndrome went into remission, and after a one-month hospital stay, he was discharged in stable condition.

Since his discharge, the patient has continued to do well, with no relapse of his nephrotic syndrome. He has maintained good compliance with his medications during outpatient visits, demonstrating the long-term effectiveness of the PFCC approach. This case highlights the importance of early identification and management of developmental disabilities in pediatric patients, particularly during emergency admissions. Through an understanding of the patient's unique needs and the involvement of the family in the care process, the healthcare team was able to provide a level of care that was both effective and compassionate.

## Discussion

The case of this 5-year-old boy with ASD highlights the critical role that PFCC plays in the management of pediatric patients with developmental disabilities, particularly during emergency admissions. Children with ASD often have heightened sensitivities and anxieties, making hospital environments particularly challenging. Traditional medical approaches, which may not take these factors into account, can exacerbate the child’s distress and lead to behavioral issues that complicate treatment.

The strategies implemented—positive phrasing, visual aids, and structured routines—are consistent with evidence showing that predictable, supportive environments reduce anxiety in children with developmental disabilities^4, 5)^. Importantly, these measures were not limited to individual providers but were implemented across the entire care team, ensuring consistency.

The PFCC approach addresses these challenges by involving the family as partners in the care process. Families provide valuable insights into the child’s behavior, preferences, and needs, which can be used to tailor medical care in a way that reduces anxiety and improves outcomes. In this case, the involvement of the family was crucial in developing strategies to help the child cope with the hospital environment.

Furthermore, the PFCC approach fosters a sense of trust and collaboration between the healthcare team and the family. Through close collaboration, the team was able to provide care that was not only medically effective but also emotionally supportive, helping to build a positive relationship with the patient and his family. This relationship is particularly important in cases involving chronic conditions or developmental disabilities, where ongoing care and support are often necessary.

## Conclusion

As the prevalence of ASD and other developmental disabilities continues to rise, adopting the principles of PFCC is becoming increasingly important for all healthcare providers, not only those in pediatric care. The case presented here demonstrates the effectiveness of the PFCC approach in managing a complex pediatric case involving ASD and nephrotic syndrome. This approach, which emphasizes collaboration, communication, and family involvement, is essential for providing high-quality care to children with special needs. As healthcare continues to evolve, the principles of PFCC will become increasingly important in ensuring that all patients, particularly those with special needs, receive the care and support they deserve.

## Author contributions

AE, AN, and MN managed the patient. AE, and AN drafted the manuscript. MS, and TS reviewed and revised the manuscript. All authors read and approved the final manuscript.

## Conflicts of interest statement

The authors declare that there are no conflicts of interest.

## Informed consent

Written informed consent for publication was obtained from the patient’s legal guardian.
